# The CXC-Chemokine CXCL4 Interacts with Integrins Implicated in Angiogenesis

**DOI:** 10.1371/journal.pone.0002657

**Published:** 2008-07-16

**Authors:** Sallouha Aidoudi, Kinga Bujakowska, Nelly Kieffer, Andreas Bikfalvi

**Affiliations:** 1 INSERM, U920, Talence, France; 2 Univ Bordeaux, Talence, France; 3 Laboratoire de Biologie et Physiologie Intégrée (CNRS/GDRE-ITI), University of Luxembourg, Luxembourg City, Luxembourg; Baylor College of Medicine, United States of America

## Abstract

The human CXC-chemokine CXCL4 is a potent inhibitor of tumor-induced angiogenesis. Considering that CXCL4 is sequestered in platelet α-granules and released following platelet activation in the vicinity of vessel wall injury, we tested the hypothesis that CXCL4 might function as a ligand for integrins. Integrins are a family of adhesion receptors that play a crucial role in angiogenesis by regulating early angiogenic processes, such as endothelial cell adhesion and migration. Here, we show that CXCL4 interacts with αvβ3 on the surface of αvβ3-CHO. More importantly, human umbilical vein endothelial cells adhere to immobilized CXCL4 through αvβ3 integrin, and also through other integrins, such as αvβ5 and α5β1. We further demonstrate that CXCL4-integrin interaction is of functional significance *in vitro*, since immobilized CXCL4 supported endothelial cell spreading and migration in an integrin-dependent manner. Soluble CXCL4, in turn, inhibits integrin-dependent endothelial cell adhesion and migration. As a whole, our study identifies integrins as novel receptors for CXCL4 that may contribute to its antiangiogenic effect.

## Introduction

Angiogenesis is the formation of new capillaries from preexisting blood vessels. Angiogenesis plays an important role in physiologic processes such as wound healing and in disease progression such as cancer, diabetic retinopathy and various inflammatory disorders [1]. In particular, the expansion of solid tumors and other cancers critically depends on angiogenesis [2], making anti-angiogenesis strategies relevant for cancer therapy [3]. CXCL4, a CXC-chemokine, is synthesized predominantly in megakaryocytes, sequestered in α-granules of platelets, and released upon activation of platelets [4]. CXCL4 and the peptide derived from its carboxyl-terminal domain (CXCL4/CTF) display a strong antiangiogenic activity *in vitro* [5]–[7] and *in vivo* [5], [7]–[8]. They suppress growth of various tumors [9]–[11] and metastasis [12] *in vivo*. This effect is related to their antiangiogenic action and not to tumor cell proliferation [8]–[12]. Although CXCL4 is one of the first agents discovered to have an antiangiogenic action in ex-*vivo* systems [5], the specific receptor mechanisms that transduce the antiangiogenic signal of CXCL4 are still poorly understood.

Angiogenesis depends on vascular endothelial cell proliferation, migration and invasion. A family of adhesion receptors known as integrin receptors tightly regulates these early angiogenic processes. Indeed, integrins are the major adhesion receptors used by endothelial cells undergoing angiogenesis to interact with their extracellular matrix (ECM). This interaction causes spreading of endothelial cells with cytoskeleton re-organization events necessary for cells to invade ECM, to proliferate, to migrate and to ultimately form new tubular vessels [13]. The integrin-dependency of tumor angiogenesis *in vivo* is evidenced by the fact that antagonists of the αvβ3 integrin, which are highly expressed in angiogenic endothelium, suppress tumor growth by inhibiting angiogenesis [14], [15]. Furthermore, the functionally and structurally homologous αvβ5 has been implicated in angiogenesis under certain conditions and selective antagonists of αvβ5 or dual antagonists of αvβ3 and αvβ5 integrins inhibit VEGF-stimulated angiogenesis *in vivo* in animal models [16]. Finally, the α5β1 integrin was shown to play a crucial role in angiogenesis and selective antagonists of α5β1 integrin block tumor angiogenesis, thereby causing regression of human tumors in animal models [17]. Several integrin inhibitors are currently tested as therapeutics for cancer [3].

Taking into account that CXCL4 is released from the α-granules of activated platelets in the vicinity of vessel wall injury [18] and that CXCL4 targets the endothelial cells *in vivo* that undergo active angiogenesis [19], [20], we examined the possibility that CXCL4 might function as a ligand for integrins. We show here that CXCL4 binds to αvβ3 and to some extent to αvβ5 and α5β1 integrins on the surface of endothelial cells. The CXCL4-integrin interaction is of functional significance, since CXCL4 modulated endothelial cell functions, such as spreading and migration through integrins. Taken together with the established importance of integrin in tumor angiogenesis, this study provides a new mechanistic context for the function of CXCL4 as an angiogenesis inhibitor.

## Results

### 1/Immobilized CXCL4 or CXCL4/CTF induces human endothelial cell spreading, and focal adhesion kinase (FAK) phosphorylation

**Figure 1:**
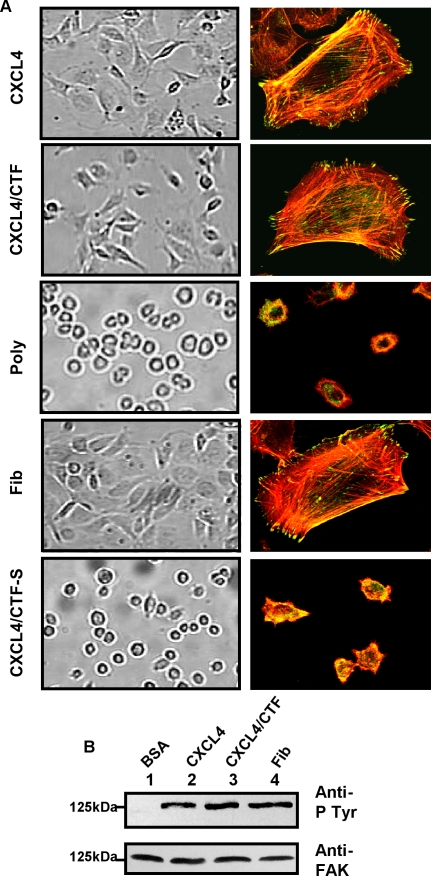
Immobilized CXCL4 or CXCL4/CTF promotes human umbilical vein endothelial cells (HUVECs) spreading, focal adhesion and stress fiber formation. (A) Analysis of spreading, focal adhesion and stress fibers. HUVECs were plated for 4h on coverslips that had been coated with (25 µg/ml) CXCL4, (25 µg/ml) CXCL4/CTF, (100 µg/ml) fibrinogen, (25 µg/ml), CXCL4/CTF-S and (100 µg/ml) poly-D-lysine. After washing, the cells are fixed. The degree of cell spreading is seen from the phase contrast micrographs (left side) Then the cells were permeabilized and stained to visualize focal adhesion (vinculin staining, green), and actin stress fibers (Rhodamin-Phalloidin staining, red) by confocal microscopy (right side). (B) Analysis of tyrosine phosphorylation of FAK. Effect of HUVEC cell adhesion to CXCL4 or CXCL4/CTF on tyrosine phosphorylation of FAK. HUVECs were maintained in suspension for 60 min (BSA, lane 1) or allowed to attach to integrins ligand (fibrinogen, lane 4) or CXCL4 (lane 2) or CXCL4/CTF (lane 3). Cells lysates containing equal amounts of protein were immunoprecipitated with anti-FAK antibody. One half of immunoprecipitates was subjected to immunoblotting with anti-phosphotyrosine mAbs, 4G10, and PY20, and the other half was probed with mAb anti-FAK.

Integrin-mediated cell attachment on cognate integrin ligands, such as ECM proteins, results in cell spreading, focal adhesion formation, and tyrosine phosphorylation of intracellular proteins [21]. When integrin inhibitors such as antibodies are immobilized on a substrate, they act as agonist and similarly activate intracellular events [22], [23]. To examine whether immobilized CXCL4 would function as an integrin agonist, HUVECs were plated on coverslips that had been coated with CXCL4. As shown in Fig. 1, immobilized CXCL4 similar to natural integrin ligands fibrinogen or fibronectin, promoted endothelial cells spreading, focal adhesion and stress fibers formation. Furthermore, to determine whether the C-terminus of CXCL4 exhibited similar effects, we used a synthetic peptide encompassing amino-acid sequence 47–70 (CXCL4/CTF). Previous data showed that the peptide retains full anti-angiogenic activity of CXCL4 [5]–[7]. As shown in Fig. 1, CXCL4/CTF demonstrated similar effects on endothelial cell spreading, focal adhesion and stress fibers formation as full length CXCL4. Furthermore, when HUVECs are plated on a –scrambled peptide containing amino acides derived from CXCL4/CTF (CXCL4/CTF-S) (that does not exhibit anti-angiogenic activity), or on polylysine (to which cells adhere in an integrin-independent manner), they remained round, and failed to spread and to induce focal adhesion formation (Fig. 1).

Focal adhesion kinase (FAK) is a 125 kDa cytoplasmic tyrosine kinase colocalised with integrins at focal adhesion contacts and rapidly becomes phosphorylated and activated upon integrin mediated cell adhesion. As shown above, immobilized CXCL4 or CXCL4/CTF promotes cell spreading with focal adhesion formation (Fig. 1A). We tested whether these events are sufficient to activate (tyrosine phosphorylation) FAK. As shown in (Fig. 1B), attachment of HUVECs to immobilized CXCL4 or CXCL4/CTF, similar to fibronectin, induced tyrosine phosphorylation of FAK. As expected, attached cell to polylysine, similar to cells in suspension, failed to tyrosine phosphorylate FAK. Taken together, these results suggest that immobilized CXCL4 or CXCL4/CTF serves as an adhesive ligand to endothelial cells and activates postligand binding events downstream of integrins.

### 2/CXCL4 or CXCL4/CTF mediates αvβ3-CHO cell adhesion through αvβ3 integrins

αvβ3 is expressed on activated endothelial cells. In addition, inhibition of αvβ3 by selective antagonists blocks angiogenesis in response to growth factors in several tumor models [24], indicating that this integrin plays an essential role in tumor growth dependent on angiogenesis. Thus, for all these reasons, we first sought to test the role of αvβ3 integrin as a putative receptor to the antiangiogenic factor, CXCL4 and its derived peptide. For this purpose, we used a CHO cells transfected with αvβ3 (αvβ3-CHO, clone A06) and mock-transfected CHO cells. First, we verified αvβ3 expression by flow cytometry using a monoclonal antibody specific for the human αvβ3 (LM609). In accordance with our previous data [*25*], αvβ3 integrin is highly expressed in αvβ3-CHO cells whereas control mock-transfected CHO cells are negative for αvβ3 expression (data not shown).

#### Immobilized CXCL4 or CXCL4/CTF supported αvβ3-CHO but not CHO adhesion, in a saturable and concentration-dependent manner

**Figure 2:**
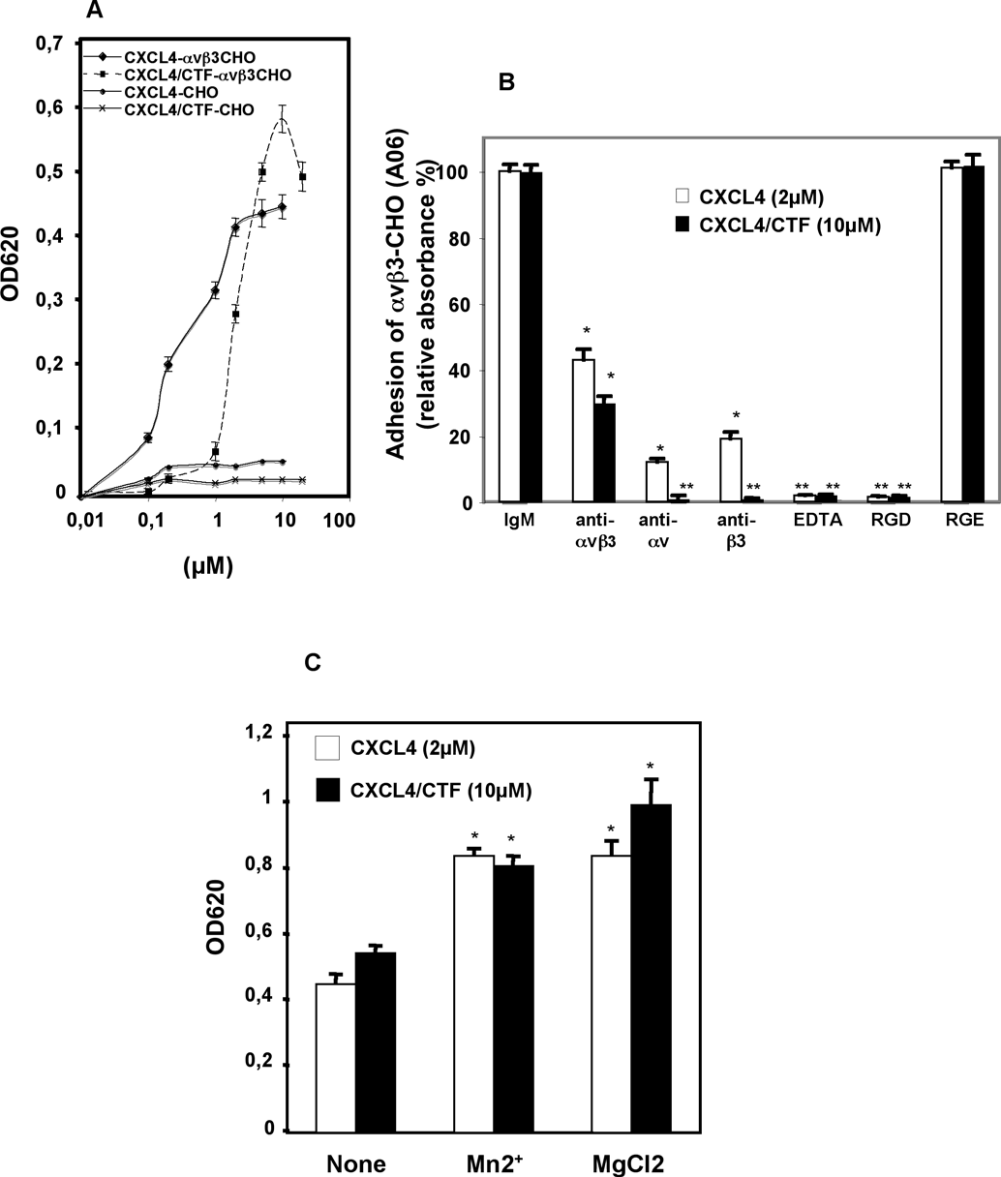
CXCL4 or CXCL4/CTF mediates αvβ3-CHO cell adhesion through αvβ3 integrins. In A. Dose dependence of αvβ3-CHO cell adhesion to CXCL4 or CXCL4/CTF. αvβ3-CHO and mock-transfected CHO were plated onto microtiter wells coated with the indicated concentrations of CXCL4 or CXCL4/CTF and cell attachment was analyzed as described in *Material and Methods*. In B, αvβ3-CHO adhesion to CXCL4 or CXCL4/CTF is αvβ3 integrin-dependent. Cells were incubated with the indicated antibodies or RGD-, RGE peptides before plating to wells coated with CXCL4 or CXCL4/CTF. Anti-integrin antibodies used were: anti-αvβ3 (LM609), anti-αv (ΑV1), anti-β3 (B3A). Cell attachment was analyzed as above. Error bars represent the mean±SD, *, P≤0.001; **, P≤0,00001 compared to CXCL4 or CXCL4/CTF in the presence of IgM control antibody; n = 4 independent experiments. In C, adhesion of αvβ3-CHO to immobilized CXCL4 or CXCL4/CTF is cation-dependent manner. The serum free medium used for the adhesion assay contained 0.25 mM of Mn2+, or 2 mM of Mg2+. Cell attachment was analyzed as above. Error bars represent the mean±SD, *, P≤0.005 compared to CXCL4 or CXCL4/CTF alone; n = 4 independent experiments.

Quantitative cell attachment assays demonstrated that immobilized CXCL4 or CXCL4/CTF supported αvβ3**-**CHO cell adhesion, but not mock-transfected CHO cells, in a saturable and concentration-dependent manner (Fig. 2A). Also, as shown in Fig 2B, adhesion to CXCL4 or CXCL4/CTF was significantly inhibited by monoclonal antibodies LM609, AV1 and B3A against αvβ3, αv and β3, respectively. These results demonstrate that αvβ3**-**CHO adhesion on immobilized CXCL4 or CXCL4/CTF is mediated by αvβ3 integrin. Consistent with these results, addition of 10 mM of EDTA, a strong inhibitor of divalent cation-dependent cell integrin receptors, blocks the adhesion of αvβ3**-**CHO cells to CXCL4 or CXCL4/CTF (Fig. 2B). Furthermore, the incubation of cells with two well established cation activators integrins, Mn2+ and Mg2+ [26], prior to adhesion of cells to immobilized CXCL4 or CXCL4/CTF enhances the cell attachment about 25% (Fig. 2C). These findings indicate that immobilized CXCL4 or CXCL4/CTF mediates αvβ3**-**CHO cell adhesion in a divalent cation-dependent manner. As with EDTA, addition of RGD-peptides blocks the adhesion of αvβ3**-**CHO to CXCL4 or CXCL4/CTF, whereas control RGE peptide had no affect on adhesion (Fig. 2B). These findings suggest that the interaction of αvβ3**-**CHO cells with CXCL4 or -CXCL4/CTF is RGD-dependent. This is consistent with the idea that αvβ3 integrin is a CXCL4 receptor in αvβ3**-**CHO cells, since αvβ3 is RGD dependent. Taken together, these results demonstrate that αvβ3**-**CHO cells attachment on immobilized CXCL4 or CXCL4/CTF is a cation-dependent process and is αvβ3 integrin mediated.

#### CXCL4 and CXCL4/CTF bind to purified integrins

**Figure 3:**
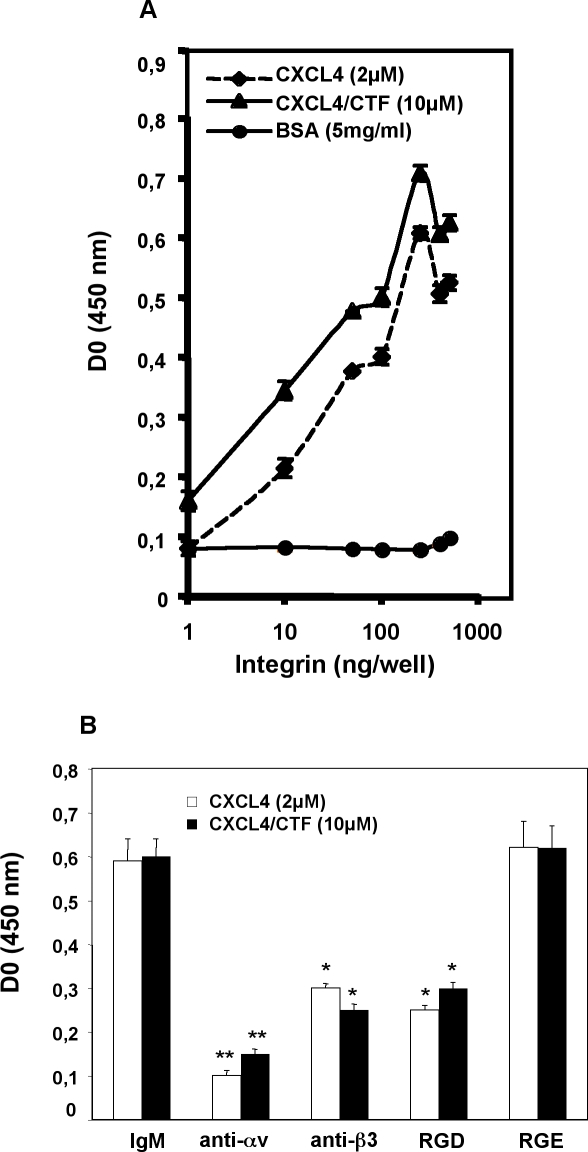
CXCL4 or CXCL4/CTF binds to purified αvβ3 integrins in a concentration-dependent and specific manners in solid-phase ligand binding assay. Α. αvβ3 integrin was added to wells coated with CXCL4 or CXCL4/CTF as indicated, and incubated overnight at 4°C. Anti-αv, peroxidase-conjugated anti-rabbit IgG antibodies, and TMB substrate were used to detect bound integrin. B. Soluble RGD-, RGE peptides or inhibitory anti-integrin antibodies were added to block the interaction between CXCL4 or CXCL4/CTF and αvβ3 integrin. Bound integrin was detected as above. Anti-integrin antibodies used were: anti-αv (AV1) and anti-β3 (B3A). Error bars represent the mean±SD, *, P≤0.005; **, P≤0,0005 compared to CXCL4 or CXCL4/CTF in the presence of IgM control antibody; n = 3 independent experiments.

To reinforce the results described above, we studied the direct interaction of CXCL4, or CXCL4/CTF, to integrin in a solid-phase ligand binding assay [27] using a purified human integrin αvβ3 **(**protein). As shown in Fig. 3A, soluble αvβ3 demonstrates a concentration- dependent and saturable binding to immobilized CXCL4 or its peptide CXCL4/CTF. The specificity of the interaction was confirmed by inhibition of CXCL4 or CXCL4/CTF binding to αvβ3 integrin with the inhibitory αv (AV1) and β3 (B3A) antibodies, as well with RGD peptides (Fig. 3B). Thus, these results demonstrate a specific and direct interaction between the angiogenic inhibitor CXCL4 or its peptide CXCL4/CTF with the αvβ3 integrin.

#### Immobilized CXCL4 or CXCL4/CTF promotes αvβ3-CHO cell spreading

**Figure 4:**
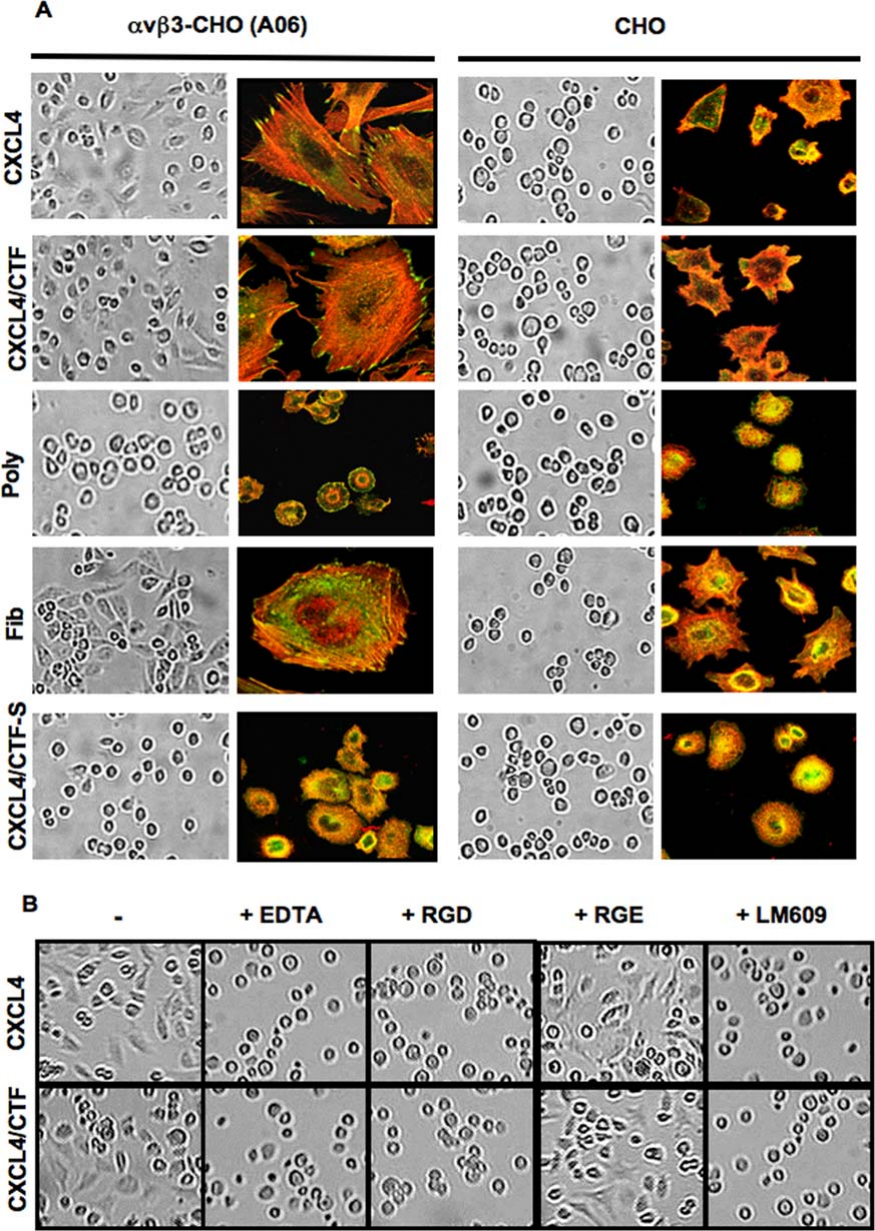
Immobilized CXCL4 or CXCL4/CTF promotes αvβ3-CHO cell spreading, focal adhesion and stress fiber formation. A. αvβ3-CHO and mock-transfected CHO cells were plated for 4 h on coverslips that had been coated with (25 µg/ml) CXCL4, (25 µg/ml) CXCL4/CTF, (25 µg/ml) CXCL4/CTF-S, (100 µg/ml) fibrinogen and (100 µg/ml) poly-D-lysine. After washing, the cells are fixed. The degree of cell spreading is seen from the phase contrast micrographs (left side). Then the cells were permeabilized and stained to visualize focal adhesion (vinculin staining, green), and actin stress fibers (Rhodamin-Phalloidin staining, red) by confocal microscopy (right side). B. Effects of function-blocking anti-integrin antibodies and EDTA and RGD-, RGE-peptides on cell spreading to CXCL4 or CXCL4/CTF. αvβ3-CHO cells were incubated with 10 mM EDTA or 25 µg/ml of RGD- or RGE-peptides or 10 µg/ml anti-αvβ3 (LM609), before plating to coverslips coated with (25 µg/ml) CXCL4 or (25 µg/ml) CXCL4/CTF for 3 h at 37°C. Wells were washed with PBS, and cells were photographed.

Confocal analysis showed that immobilized CXCL4 or CXCL4/CTF promoted αvβ3-CHO cell spreading, focal adhesion and stress fibers formation (Fig. 4). These events were blocked in presence of EDTA or RGD peptides or with antibodies against αvβ3. In contrast, mock-transfected CHO cells did not appreciably spread and generate stress fibers of actin on CXCL4 or CXCL4/CTF. In addition, when αvβ3-CHO cells are plated on a scrambled peptide derived (CXCL4CTF-S), or on polylysine, they remained round, and failed to spread and to induce focal adhesion formation (Fig. 4). These results indicate that immobilized CXCL4 or CXCL4/CTF induces αvβ3-CHO cell spreading through αvβ3 integrins. Collectively, these results show that CXCL4 or CXCL4/CTF interacts with αvβ3 integrins and activates postligand binding event downstream to integrins, such as cell spreading.

### 3/Human endothelial cells (HUVECS) adhere on immobilized CXCL4-, or CXCL4/CTF- through αvβ3, and additional integrins, αvβ5 and α5β1

**Figure 5:**
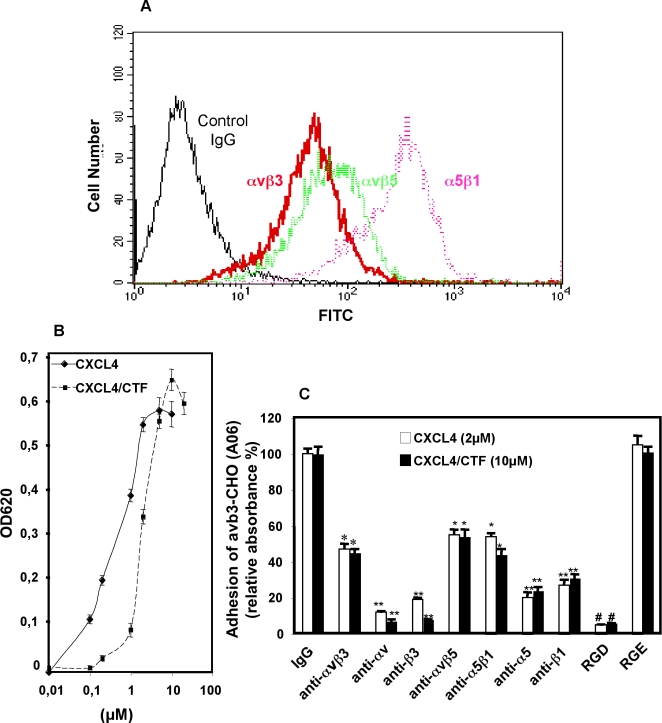
CXCL4-, or CXCL4/CTF-integrin mediates HUVECs adhesion through endogen αvβ3, αvβ5 and α5β1 integrins. A. Expression profile of integrins in HUVECs cells analyzed by flow cytometry. HUVECs were incubated with primary antibodies specific for αvβ3 (LM609), or αvβ5 (P1F6), or α5β1 (HA5) and antibody binding was detected with FITC-labeled secondary antibody as described under *Materials and Methods*. Cells stained with secondary antibody (2° Ab) only were used as a negative control. B. Dose dependence of HUVECs cell adhesion to CXCL4 or CXCL4/CTF. HUVECs were plated onto microtiter wells coated with the indicated concentrations of CXCL4 or CXCL4/CTF and cell attachment was analyzed as described in *Material and Methods* C. Cell adhesion to CXCL4 and CXCL4/CTF is αvβ3, αvβ5 and α5β1 integrins dependent. Cells were incubated with the indicated antibodies or RGD-, RGE peptides before plating to wells coated with CXCL4 or CXCL4/CTF. Anti-integrin antibodies used were: anti-αvβ3 (LM609), anti-αv (ΑV1), anti-β3 (B3A), anti-αvβ5 (P1F6), anti-α5β1 (JBS5), anti-α5 (P1D6) and anti-β1 (CD29). Cell attachment was analyzed as above. Error bars represent the mean±SD, *, P≤0.001; **, P≤0,0001; #, P≤0,00001 compared to CXCL4 or CXCL4/CTF in the presence of IgM control antibody; n = 4 independent experiments.

To investigate the interaction of CXCL4 or CXCL4/CTF with endogenous integrins, we tested the interaction of CXCL4 or CXCL4/CTF with integrins expressed on the surface of cultured HUVECs. We first screened the expression of endogenous integrins using a flow cytometry analysis. Accordingly with previous data [28], we found that αvβ3, αvβ5 and α5β1 were constitutively expressed on HUVECs cells using anti-human integrin antibodies specific for human integrin antibodies specific for αvβ3 (LM609), αvβ5 (P1F6), and α5β1 (HA5) (Fig. 5A). We next studied whether αvβ3, αvβ5 and α5β1 integrins expressed on HUVECs could bind to CXCL4 or CXCL4/CTF. As shown in Fig. 5B, immobilized CXCL4 or CXCL4/CTF supported HUVECs cell adhesion in a saturable and concentration-dependent manner. Adhesion to CXCL4 or CXCL4/CTF was blocked in presence of RGD peptides or with antibodies against αvβ3, thus confirming as above, that human endothelial cells adhere to immobilized CXCL4 or CXCL4/CTF through αvβ3 integrins in an RGD dependent manner (Fig. 5C). Interestingly, blocking antibodies against αvβ5 (P1F6) and α5β1 (JBS5), as with αvβ3, significantly reduced the adhesion of HUVECs cells on immobilized CXCL4 or CXCL4/CTF (Fig. 5C). In contrast, blocking antibody against α2β1-integrin did not affect cell adhesion on immobilized CXCL4 or CXCL4/CTF (data not shown). Thus, these results demonstrate that endothelial cells adhere to immobilized CXCL4 or CXCL4/CTF through αvβ3, and other integrins, αvβ5 and α5β1, which play a crucial role in angiogenesis.

### 4/Integrin agonists, Mn2+ and PMA, enhance cell spreading, with focal adhesion and stress fibers formation, on immobilized CXCL4 or CXCL4/CTF

**Figure 6:**
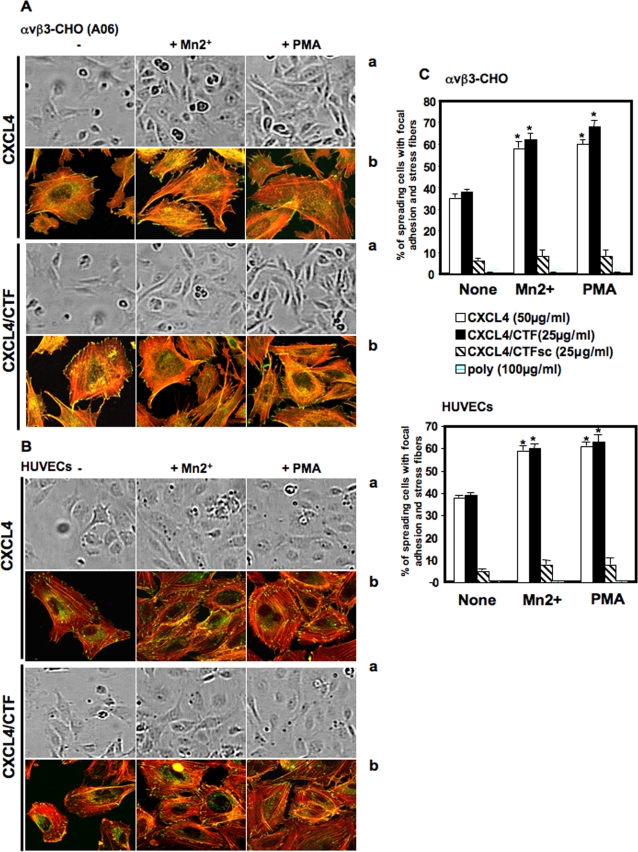
Integrin agonists, Mn2+ and PMA, enhance cell spreading, with focal adhesion and stress fiber formation, on immobilized CXCL4 or CXCL4/CTF A, B. a. Phase contrast analysis show an enhance of the number of αvβ3-CHO and HUVECs spreading on immobilized CXCL4 or CXCL4/CTF, upon stimulation with Mn2+ or PMA, b. Confocal analysis show an increase of the number of cell spreading with focal adhesion (Vinculin staining, green) and stress fibers (Rhodamin-Phalloidin staining, Red) formation in αvβ3-CHO and HUVECs spread on immobilized CXCL4 or CXCL4/CTF treated with integrin stimulators, Mn2+ or PMA, as compared with no agonists. C. 100 adherent cells on different substrat were scored for focal adhesions and stress fibers by two independent observers. Data are the means±SEM of 4 separate experiments. Error bars represent the mean±SD, *, P≤0.005 compared to CXCL4 or CXCL4/CTF in the absence of integrin agonists.

To further examine the postligand events downstream to integrins induced by immobilized CXCL4 or CXCL4/CTF, we examined in more detail cell spreading. As shown above, confocal analysis shows that immobilized CXCL4 or CXCL4/CTF promoted cell spreading, focal adhesion and stress fibers formation (Fig. 1,4). When the number of cells spread on different immobilized substrates was quantified, we found that immobilized CXCL4 or CXCL4/CTF promoted cell spreading by about 50 to 60% in the absence of integrin agonists. When we analyzed the spreaded cells by confocal microscopic we found that around 40% of these cells spread on CXCL4 or CXCL4/CTF (versus 60% with fibronectin, data not shown) presented focal adhesions with stress fiber formation (Fig. 6). These events were significantly increased (from 40% to 60%) when we added two-well known integrin activators, extracellular (Mn2+) and intracellular (Phorbol 12-Mryristate 13 Acetate (PMA) agonists (Fig. 6). These agonists activate αvβ3 integrin (convert αvβ3 to a high-affinity conformation) and stimulate αvβ3 functions [29], such spreading cells, as well as that of other integrins [30], [31]. Incubation of cells with 250 µM of Mn2+ or 100 nM of PMA produced an increase in both the number of attached cells and in cell spreading of about 25% and 30% respectively (Fig. 6). This enhancement was completely blocked by adding EDTA or RGD (data not shown). This finding suggests that the interaction of CXCL4 or CXCL4/CTF to integrins, similar to natural integrin ligands, is subject to integrin function regulation such as affinity modulation (*see* *Discussion*).

### 5/The ability of CXCL4 to bind to integrin is related to its anti-angiogenic action

The scrambled control peptide CXCL4/CTF-S fails to inhibit endothelial cell proliferation [32]. We thus tested whether this inactive peptide could bind to integrins. We found that, in contrast with CXCL4/CTF and similar to polylysine, αvβ3-CHO or HUVECs plated on immobilized CXCL4/CTF-S peptide did not adhere significantly. Cells remained round and failed to assemble focal adhesion and to induce stress fibers (Fig. 1,4). The addition of the two integrin activators, Mn2+ and PMA, had no effect on the number of attached cells or on cell spreading on PF-4/CTF-S as shown by confocal analysis (Fig. 6C). These results suggest that the ability of CXCL4 and CXCL4/CTF to bind integrins is correlated with their anti-angiogenic activity.

### 6/Soluble CXCL4 or CXCL4/CTF inhibits cell adhesion to immobilized fibronectin and vitronectin

To investigate whether CXCL4 or CXCL4/CTF could inhibits cell adhesion to integrin ligands, we tested the effect of soluble CXCL4 or CXCL4/CTF on αvβ3-CHO and HUVECs cells adhesion to specific αvβ3 and α5β1 ligands, vitronectin and fibronectin respectively. We found that the pre-incubation of cells with soluble CXCL4 or CXCL4/CTF induces cell rounding, and significantly inhibits cell adhesion to immobilized fibronectin and vitronectin, in a concentration-dependent manner (Fig. S1). CXCL4 or CXCL4/CTF did not affect cell adhesion to collagen I (data not shown). These results suggest that soluble CXCL4 and CXCL4/CTF act as antagonists of αvβ3 and α5β1 integrins to their ligands.

### 7/immobilized CXCL4 or CXCL4/CTF promotes and soluble CXCL4 or CXCL4/CTF inhibits endothelial cell migration in an integrin-dependent and -specific manners

**Figure 7:**
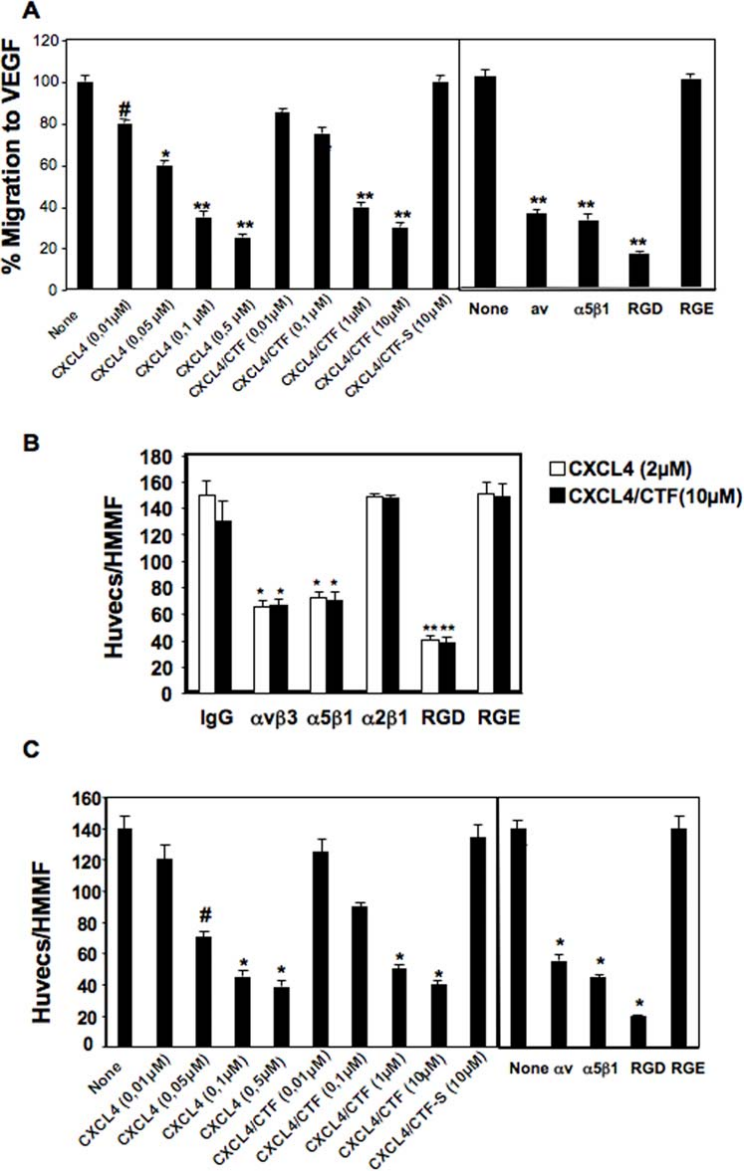
Immobilized CXCL4 or CXCL4/CTF promotes and soluble CXCL4 or CXCL4/CTF inhibits integrin-dependent endothelial cell migration. A. Soluble CXCL4 or CXCL4/CTF inhibits migration on fibronectin toward VEGF (5 ng/ml). HUVECs migration was determined in the presence or absence of the indicated concentrations of CXCL4 or CXCL4/CTF or CXCL4/CTF-S or 25 µg/ml of RGD-, RGE peptides or 10 µg/ml of anti-integrin antibodies (anti-αv; AV1 and anti-α5β1; JBS5) as described in *Material and Methods*. Relative cell migration is indicated, each value is a mean±SEM from 3 independent experiments. Error bars represent the mean±SD, ^#^, P≤0.05; *, P≤0.005, **, P≤0.001 compared to the number of cell migrated in the absence of CXCL4 or CXCL4/CTF or anti-integrin antibodies or RGD peptides. B. Immobilized CXCL4 or CXCL4/CTF supports HUVECs migration in αvβ3 and α5β1 integrins-dependent manner. Cell migration assay was performed on immobilized CXCL4 or CXCL4/CTF in serum free medium and in the presence or absence of inhibitory anti-integrin antibodies or RGD-, RGE peptides. The results at each point are the mean cell number of 10 randomly selected high magnification microscopic fields. Error bars represent the mean±SD, *, P≤0.005; *, P≤0.001 compared to CXCL4 or CXCL4/CTF in the presence of IgG control antibody; n = 3 independent experiments. C. Soluble CXCL4 or CXCL4/CTF inhibits migration on fibronectin. HUVECs migration was determined in serum free medium and in the presence or absence of the indicated concentrations of CXCL4 or CXCL4/CTF or CXCL4/CTF-S or inhibitory anti-integrin antibodies or RGD-, RGE peptides. Relative cell migration is indicated, each value is a mean±SEM from 3 independent experiments. Error bars represent the mean±SD, ^#^, P≤0.005; *, P≤0.001 compared to the number of cell migrated in the absence of CXCL4 or CXCL4/CTF or anti-integrin antibodies or RGD peptides.

Cell migration is an essential step in the angiogenesis process [13]. Previous studies showed that CXCL4 or CXCL4/CTF block endothelial cell migration in response to FGF-2 or VEGF in stimulation *in vitro* [7], [33], a process thought to mediate the anti-angiogenic effect of CXCL4 *in vivo*. On the other hand, αvβ3 [34], and α5β1 [17] integrins have a crucial role in cell migration during angiogenesis. Taking into account our findings, we determined whether CXCL4 and its antiangiogenic derived peptide modulates endothelial cell migration through αvβ3 and/or α5β1 integrins. Our finding showed that soluble CXCL4 or CXCL4/CTF inhibited HUVECs cells migration through fibronectin a reported αvβ3 and α5β1 ligand [13], toward VEGF, in a dose depending manner (Fig. 7A). This migration is similarly inhibited by adding RGD peptides or by the blocking integrin antibodies, anti-αvβ3 LM609 or anti-α5β1 JBS5 (Fig. 7A). These results are consistent with previous data [7], [33], and suggest that CXCL4 or CXCL4/CTF inhibits endothelial cell migration in an integrin dependent manner.

To study the functional significance of the specific CXCL4-integrin interaction, we examined the capacity of CXCL4 to modulate cells functions under conditions in which these functions are strictly dependent on integrins, and not any other agents. For this purpose, we use a haptotactic Boyden chamber assay to assess cell migration in the absence of growth factors, or other soluble chemoattractants (such as VEGF). Using this assay, previous data showed that immobilized integrin inhibitors act as functional agonists and similarly promote integrin functions as cell migration [22]. On the other hand, cell migration is efficiently inhibited by the same integrin inhibitors when given in solution to the cells [22]. We demonstrated that CXCL4 or CXCL4/CTF is similarly capable of modulating endothelial cell migration. Thus, HUVEC cells under serum free-medium migrated in a haptotactic Boyden chamber assay through a CXCL4- or CXCL4/CTF-coated membrane and this migration was significantly inhibited by RGD peptides or by the blocking integrin antibodies, anti-αvβ3 LM609 or anti-α5β1 JBS5 (Fig. 7B). In control experiments, blocking antibody against α2β1-integrin did not affect cell migration through CXCL4- or CXCL4/CTF-coated membrane (Fig. 7B). Soluble CXCL4 or CXCL4/CTF, in turn, interferes with integrin-dependent cell migration. As shown in (Fig. 7C), soluble CXCL4 or CXCL4/CTF, but not the inactive peptide CXCL4/CTF-S, inhibited cell migration on immobilized fibronectin, in a concentration-dependent manner. In control experiments, RGD peptides or blocking antibodies against αv or α5β1−integrin inhibited endothelial cell motility on fibronectin (Fig. 7C). Soluble CXCL4 and CXCL4/CTF, or antibodies against αv and α5β1 integrins, did not affect cell migration on collagen I, on which the cells migrated in a manner dependent on non-RGD binding β1 integrins (data not shown). Thus, immobilized CXCL4 or CXCL4/CTF promotes and soluble CXCL4 or CXCL4/CTF inhibits endothelial cell migration in an integrin-dependent and -specific manners.

## Discussion

We sought to assess whether CXCL4, a CXC chemokine that exhibit potent anti-angiogenic activities, and its C-terminus derived peptide CXCL4/CTF, would function as ligands for the integrin receptors on the surface of endothelial cells. We report here (**1)** that CXCL4 or CXCL4/CTF binds to αvβ3 and to some extent to αvβ5 and α5β1 integrins. (**2)** CXCL4 or CXCL4/CTF binding to integrins on the surface of αvβ3-CHO and HUVECs is blocked by RGD peptide but not by RGE peptide. (**3)** The inactive peptide derived from CXCL4/CTF (CXCL4/CTF-S), fails to interact with integrins on the surface of αvβ3-CHO or HUVECs. (**4**) Immobilized CXCL4 or CXCL4/CTF promotes αvβ3-CHO and HUVEC cells spreading, focal adhesion and stress fibers formation and FAK phosphorylation (**5)** Functional studies show that immobilized CXCL4- or CXCL4/CTF support endothelial cells migration in an integrin-dependent manner and soluble CXCL4 or CXCL4/CTF, in turn, inhibits integrin-dependent endothelial cell migration.

Thus, the present study is the first to show that CXCL4 interacts with integrins. This interaction may constitute a novel mechanism for the inhibitory effects of CXCL4 on angiogenic blood vessels.

Several lines of cell biological and biochemical evidence demonstrated the interaction between CXCL4 and integrins in our study. Thus, inhibitory anti-integrin antibodies prevented αvβ3-CHO and HUVEC cell attachment whereas CXCL4 interacted directly with purified αvβ3 integrin in a specific manner in a solid-phase binding assay. Despite the fact that neither CXCL4 nor CXCL4/CTF contain an RGD sequence, the interaction of CXCL4 with integrins is divalent cation-dependent and is blocked by RGD peptide. Interestingly, a number of endogenous angiogenesis inhibitors such as endostatin [27], arresten [35] canstatin [36] and tumstatin [37], [38] also bind directly to integrin, despite the fact that they lack the RGD-binding site. For example, the interaction of endostatin with α5β1 integrin is inhibited by RGD peptide, however tumstatin binds to αvβ3 in an RGD-independent manner. The fact that the interaction of CXCL4 with integrins is blocked by the RGD peptide indicates that CXCL4 interacts with the same or an overlapping RGD-binding site in integrin. Alternatively, it is possible that other amino acid sequences within CXCL4 also interact with the RGD binding site. Interestingly, CXCL4 and CXCL4/CTF both contain the NGR sequence that is known to interact with integrins, such as αvβ3, αvβ5 and α5β1 [39], [40]. The NGR motif homes to the tumor vasculature, but not in normal endothelium, through its interaction with integrins. As a consequence, when coupled to anticancer drugs, the NGR peptide was shown to enhance their *in vivo* anti-tumor action and to reduce their toxicity [41]. Additional studies are required to determine the sequence motif of CXCL4 that integrins recognize.

Our results show that immobilized CXCL4 or CXCL4/CTF supported cell spreading, focal adhesion and stress fibers formation and tyrosine phosphorylation (i.e., activation) of the focal adhesion kinase (FAK). These events are specific to integrin signaling [21]. This indicates that immobilized CXCL4 or CXCL4/CTF activates postligand binding events downstream of integrins. The addition of two well-known integrin activators, Mn2+ -acting as integrin activator from outside the cells (referred to as outside/in signaling)- and PMA -activating integrins from inside the cells via activation of PKC (referred to as inside/out signaling)-, produced a strong increase in both the number of attached cells and cell spreading on immobilized CXCL4 or CXCL4/CTF, comparable to fibrinogen. Thus, PF4 seems to bind to αvβ3 integrins as a natural ligand of αvβ3, such as fibrinogen, which binds with low affinity to unstimulated αvβ3 and binds with high affinity to activated αvβ3 upon stimulation by agonists, such as Mn2+ or PMA [29]. Thus, our finding suggests that interaction of CXCL4 or CXCL4/CTF to integrins is subject to integrin function regulation (i.e. affinity modulation). These results may be relevant to the antiangiogenic activity of CXCL4 in some pathophysiological circumstances in vivo. Indeed, it was shown that the basal activation state of αvβ3 varies with the cell type and that an increase in the number of active αvβ3 with high affinity for their ligands is correlated with the acquisition of cell malignancy [29] and with the metastasis of some cancer, such as in human breast cancer [42]. Considering that the binding of CXCL4 to integrins depends on the integrin state (active or not), our results suggest that the binding of CXCL4 to endothelial cells should increase during pathological angiogenesis, where affinity modulation of integrins occurs.

Our results show that soluble CXCL4 or CXCL4/CTF inhibited endothelial cell adhesion to immobilized matrix proteins, as fibronectin or vitronectin. These results suggest that soluble CXCL4 or CXCL4/CTF act as antagonist for integrins αvβ3 and α5β1, to mediate its inhibition effect on -endothelial cells adhesion- angiogenesis. Theoretically, it is possible that CXCL4 could also act on attached endothelial cells by further promoting spreading. However, we clearly demonstrated that surface immobilized but not soluble CXCL4 promoted spreading. This finding argues against a similar effect on already attached cells. In addition, it is unlikely that spreading of already attached endothelial cells is inhibited by CXCL4, because of the strong binding of integrins to matrix proteins.

Although α5β1 is the most abundant integrin on HUVECS (Fig. 5A), adhesion experiments on immobilized CXCL4 in the presence of different blocking antibodies against αvβ3, α5β1, β1, α5, αv, β3 may suggest a greater interaction of CXCL4 to αvβ3 integrin (Fig. 5C). However this conclusion must be interpreted with caution because these differences may also be due to more efficiency antibody binding to αvβ3 than to α5β1. Also, a quantitative analysis comparing affinities is required to ascertain the potentially better interaction of CXCL4 to αvβ3. Nevertheless, assuming that CXCL4 interacts better with αvβ3 than α5β1, we may speculate that CXCL4 contains additional sequences for αvβ3 interaction. The expression of both integrins αvβ3 and α5β1 are significantly up-regulated on endothelial during angiogenesis. While α5β1 selectively recognizes primarily a single ECM protein ligand, fibronectin, αvβ3 can bind several ligands such as vitronectin, fibronectin, fibrinogen, and other matrix proteins (13). The fact that CXCL4 may be a better ligand for αvβ3 than α5β1, thus blocking the interaction of αvβ3 integrin to its various ligands, may increase its inhibitory effect on angiogenesis.

To study the functional significance of the CXCL4-integrin interaction on angiogenesis, we examined the capacity of CXCL4 to modulate endothelial cell migration. We found that CXCL4 modulates endothelial cell migration, in an integrin-dependent and -specific manners. Cell migration is a vital step in the angiogenesis process and is governed by pro-angiogenic factors, such as VEGF and FGF, and integrins [13], [43]. Angiogenic stimulus by growth factors induces an increased expression of integrins in endothelial cells undergoing angiogenesis, thereby leading cells to spread, to migrate and to ultimately form new tubular vessels [13]. Previous studies showed that both CXCL4 and CXCL4/CTF block endothelial cell migration in response to VEGF or FGF growth factors [7], [33]. This action is believed to mediate the anti-angiogenic effect of CXCL4, although its receptor mechanism was not characterized. Here we report that soluble CXCL4 or CXCL4/CTF, but not the inactive peptide CXCL4/CTF-S, inhibits endothelial cell migration in αvβ3 and α5β1 in an integrin-dependent and -specific manners. Similarly, endostatin and thrombospondin-1, two endogenous angiogenesis inhibitors, inhibited endothelial cell migration through β1 integrins [27], [44]. Our results suggest that integrins serve as functional receptors for CXCL4 to mediate its inhibition effect on -endothelial cells migration- angiogenesis. On the other hand, other data indicate that VEGF directly activates integrins and enhance cell migration mediated by αvβ3 and α5β1 [34] Furthermore, FGF-2 stimulated endothelial cell migration mediated by αvβ3 [45]. Further studies are needed to determine the potential significance of integrin-growth factor receptor cross-talk in CXCL4 action.

Previous studies showed that CXCL4 and its derived peptide CXCL4/CTF inhibit angiogenesis by interfering with the angiogenic effect of the growth factors FGF and VEGF165, either by directly binding to these angiogenic factors, thereby blocking their interaction with their specific receptors [32], [46]–[47], or by competing with the binding of FGF or VEGF to heparan sulfate proteoglycans on the cell surface [46], [48]. Furthermore, CXCL4 was recently shown to bind to CXC3B, a chemokine receptor isoform that is present in some vascular beds [49]. However, the role of CXC3-B receptors in the anti-angiogenic function of CXCL4 remains to be fully established [50]. Our study suggests a novel additional mechanism for the antiangiogenic effects of CXCL4, which involves direct targeting of vessel through integrins. Since integrins are overexpressed in endothelial cells undergoing angiogenesis, this may explain why, in vivo, CXCL4 tends to preferentially target angiogenic blood vessels [19], [20]. Furthermore, this targeting mechanism could be useful for selective treatments of cancer by CXCL4. Indeed and as mentioned above, peptides were shown to home selectively in tumor vasculature, through their interaction with integrins, and when coupled to anticancer drugs, they enhanced the *in vivo* anti-tumor action and reduced the toxicity of anticancer drugs [41]. Finally, integrin-binding of CXCL4 or CXCL4/CTF is also consistent with a heparan sulfate proteoglycan-independent mechanism. Indeed, VEGF121, an endothelial cell mitogen that lacks heparin affinity, is inhibited by CXCL4 [47]. CXCL4 also antagonizes EGF-mediated endothelial cell proliferation independently from glycosaminoglycans [51].

In summary, a general picture of CXCL4's antiangiogenic mechanisms is emerging from the results described above. CXCL4 inhibits neovascularization *in vivo* by several distinct, although not necessarily exclusive, mechanisms including direct integrin binding– reported here –, direct interaction with angiogenic molecules [32], binding to proteoheparan sulfate–which acts as low affinity co-receptors for growth factors, such as FGF–[46], [48] or activation of CXCR3-B [49]. These different mechanisms may operate in parallel and in function of the site and/or the type of vessel that undergo angiogenesis. Additional studies will be required to fully understand the significance of integrin binding and other mechanisms involved in CXCL4's antiangiogenic activity *in vivo*.

## Materials and Methods

### Reagents and Cell Culture

Fibronectin from human, plasma Fibrinogen from human plasma, poly-D-lysine and bovine serum albumin (BSA, fraction V) were purchased from Sigma. RGDS and RGES peptides were from Bachem, human purified platelet factor 4/CXCL4 (CXCL4) was obtained from Hyphen BioMed, France. C-terminal peptides of CXCL4, CXCL4/CTF (NGRKICLDLQAPLYKKIIKKLLES) or CXCL4/CTF-S (LGLKPLKQELIAYRDNK IKSICLK) were purchased from ThermoHybaid (Ulm, Germany). Purified integrins and the purified monoclonal anti-human integrin antibodies LM609 (anti-αvβ3), P1F6 (anti-αvβ5), JBS5 and HA5 (anti-α5β1), B3A (anti-β3), AV1 (anti-αv), CD29 (anti-β1), P1D6 (anti-α5) were purchased from Chemicon. Mouse Ig1 antibody was purchased from Chemicon. Peroxidase and FITC (Fluorescein isothiocyanate) conjugated goat anti-mouse immunoglobulins were purchased from DAKO. Mouse monoclonal anti-human vinculin antibody was from Sigma and Rhodamin-Phalloidin from Molecular probes. The Chinese hamster ovary (CHO) cell line CRL9096, as well as CHO clones expressing human αvβ3 integrin (αvβ3-CHO, A06) were established as described (25). Cells lines were grown at 37°C, 5% CO2 in Dublecco's Modified Eagle Medium (GIBCO) supplemented with 10% foetal bovine serum, 2 mM glutamine, penicillin and streptomycin (100 UI/ml) and 1% non-essential amino acids. Human umbilical vein endothelial cells (HUVECs, Clonetics) were cultured in EGM medium (Cambrex) supplemented with 10% foetal bovine serum, 2 mM glutamine, and penicillin and streptomycin (100 UI/ml). Experimentation was carried out at cell passage number 4–10.

### Flow Cytometry

Analysis cell-surface integrin expression was performed as described [29]. Cells were suspended in incubation Walsh buffer (137 mM NaCl, 2.7 mM KCL, 3.3 mM NaH_2_PO_4_, 3.8 mM HEPES, 1 mM MgCl2, 5.5 mM glucose, and 1 mg/ml BSA, pH 7.4) and 2×10^5^ cells incubated for 30 min on ice with a monoclonal antibody (10 µg/ml) specific for αvβ3 (LM609), or αvβ5 (P1F6), or α5β1 (HA5). After washing, the cells were incubated for 20 min on ice with FITC-conjugated goat anti-mouse IgG, washed again, and analyzed on a FACSCalibur flow cytometer (Becton Dickinson Biosciences). As a negative control, samples were incubated with the secondary antibody alone.

### Cell Attachment Assay

Cell attachment was assayed as described [52]. Microtiter wells (Immulon 2, ThermoLabsystems) were coated for 2 h at 37°C with the indicated concentrations of proteins. The wells were blocked for 1 h with 1% BSA in PBS. Cells were briefly trypsinized followed by washes with serum-containing medium and serum-free medium containing 0,5% BSA. Cells were suspended in 3,5×10^5^ cells/ml in serum-free medium containing 0,5% BSA and incubated in the presence or absence of 10 mM EDTA, 25 µg/ml RGD, RGE peptides or 10 µg/ml of anti-integrin antibodies for 15 min at room temperature. To inhibit adhesion on various ECMs proteins with soluble CXCL4 or CXCL4/CTF, CXCL4 or CXCL4/CTF were incubated with cells 30 min in suspension prior plating onto wells, as indicated in the figures. 100 µl of cells suspensions were added to the coated wells, and the plates were incubated at 37°C for 60 min. Non attached cells were removed by washing with PBS and then fixed and stained in 30% methanol, 10% acetic acid, containing 0,1% Coomassie blue. After extensive washing, the cells were lysed in 1% SDS, after which the amount of lysed cells were quantified by measuring their absorbance at 620 nm. Background absorbance observed in the wells coated with BSA was deducted from the values obtained. Each time point represented three independent experiments performed in triplicate.

### Solid-Phase Ligand-Binding Assay

Solid-phase ligand binding assay was performed as described previously with minor modifications [27]. Immulon 2 microtiter wells were coated with 25 µg/ml CXCL4 or CXCL4/CTF in PBS overnight at 4°C. The wells were blocked with 1% BSA in walsh buffer at room temperature for 1 h. Octylglucoside αvβ3 integrin was added on coated wells and incubated for 2 h at 37°C. Unbound integrin molecules were extensively washed with walsh buffer containing 0,05% Tween 20. The bound integrin molecules were incubated with 1/1000 dilution of a polyclonal anti-αv cytoplasmic domain antibody (Chemicon) for 1 h at room temperature. After extensive washes with walsh buffer-0,05% Tween 20, the bound antibodies were detected by using Peroxidase-Conjugated goat anti-rabbit IgG (Dako). TMB liquid substrate (sigma) was added to the wells, the reactions were stopped with 0,5 M H_2_SO_4_, and absorbance was measured at 450 nm. Background absorbance in the wells coated with BSA was deducted from the values obtained.

### Cell Spreading and Confocal Microscopy Analysis

Cells were washed twice with serum-free medium, suspended to the serum-free medium, and plated on coverslips that had been coated with (25 µg/ml) CXCL4, (25 µg/ml) CXCL4/CTF, (25 µg/ml) CXCL4/CTF-S, (100 µg/ml) fibrinogen and (100 µg/ml) poly-D-lysine and incubated at 37°C for 4 h. After removing nonadherent cells, the adherent cells were fixed in 4% parafolmaldehyde, permeabilized with 0,2% Triton X-100 in PBS, and stained with monoclonal anti-vinculin antibody, FITC-anti-mouse IgG and rhodamine-phalloidin [53]. Cells were analyzed by confocal laser scanning microscopy and photomicrographs were prepared using Adobe Photoshop 8.

### Analysis of Tyrosine Phosphorylation of FAK

Tyrosine Phosphorylation of FAK was assayed as described [54]. HUVECs were plated for 60 min on dishes that had been coated with 5 mg/ml of BSA or (100 µg/ml) fibrinogen or (25 µg/ml) CXCL4 or (25 µg/ml) CXCL4/CTF or (100 µg/ml) poly-D-lysine. After 60 min, non-adherent cells from BSA-coated plates were diluted 1:1 with PBS, sedimented at 100 g for 5 min and washed once with PBS before lysis in complete RIPA buffer (1% Triton X-100, 1% sodium deoxycholate, 0.1% SDS, 158 mM NaCl, 10 mM Tris, pH 7.4, 1 mM Na_2_EGTA, 1 mM sodium vanadate, 0.5 mM leupeptin, 0.25 mg/ml pefabloc, 5 mg/ml aprotinin). The adherent cells from plates coated with fibrinogen or CXCL4 or CXCL4/CTF were rinsed twice with PBS, lysed on the plates with ice-cold complete RIPA buffer and scrapped into microcentrifuge tubes. Lysates were incubated for 30 min on ice and clarified supernatants for immunoprecipitation. Equal amounts of protein from each lysate (200 µg of protein) were immunoprecipitated with rabbit antiserum specific for FAK (Santa Cruz). One half of immunoprecipitates were subjected to western blotting with anti-phosphotyrosine mAbs, 4G10 (BD Transduction Laboratories) and PY20 (Upstate Biotechnology), and the other half was probed with mAb anti-FAK (BD Transduction Laboratories). Immunoreactive bands were detected by enhanced cheminuluminescence detection.

### Endothelial cell Migration

Haptotactic cell motility was measured by using a modified Boyden chamber (BD, Falcon) as previously described [22]. The undersurface of the membrane filter was coated with 25 µg/ml of CXCL4 or 25 µg/ml CXCL4/CTF or various matrix proteins. After washing the membranes with PBS, nonspecific adhesion sites were saturated with 1% BSA at 22°C for 1 h. HUVECs (3×10^4^) were added to the upper chambers in serum-free medium and were allowed to migrate to the underside of the chamber for 4 h at 37°C. Cell migration was measured after 4 h of incubation at 37°C. All non migrated cells were removed from the upper face of the membrane filter with the cotton swab and cells migrated to the lower face were fixed and stained in 30% methanol, 10% acetic acid, containing 0,1% Coomassie blue. Subsequently, the number of stained migrated cells (to the lower face of the membrane filter) was counted. Migration results are expressed in terms of the average number of cells/high-magnification microscopic field. To inhibit migration on CXCL4 or CXCL4/CTF, cells with pretreated with 10 µg/ml of anti-integrin antibodies for 30 min in suspension prior to plated in chambers. To inhibit migration on various matrix proteins with soluble CXCL4 or CXCL4/CTF, CXCL4 or CXCL4/CTF were incubated with cells 30 min in suspension prior adding to the upper chambers, as indicated in the figures. In chemotactic cell motility experiments, VEGF (5 ng/ml) was added in the lower chambers. To inhibit migration on fibronectin to VEGF with soluble CXCL4 or CXCL4/CTF, CXCL4 or CXCL4/CTF were incubated with cells 30 min in suspension prior adding to the upper chambers. In all experiments, nonspecific migration was determined using BSA as a ligand and was subtracted from the values of relative cell migration obtained on different matrix proteins.

### Statistical Analysis

Student's test was used to determine the significance of differences.

## Supporting Information

Figure S1 Soluble CXCL4 or CXCL4/CTF inhibits cell adhesion to fibronectin and vitronectin. HUVECs and αvβ3-CHO adhesion on immobilized (10 µg/ml) fibronectin or (10 µg/ml) vitronectin was determined in the presence or absence of the indicated concentrations of CXCL4 or CXCL4/CTF or CXCL4/CTF-S as described in Material and Methods. Error bars represent the mean+SD, #, P<0.005; *, P<0.001 compared to the cell adhesion in the absence of CXCL4 or CXCL4/CTF; n = 2 independent experiments.
(39.08 MB DOC)
