# Supplementary figures and images for: The CXC-Chemokine CXCL4 Interacts with Integrins Implicated in Angiogenesis
Source: PLoS One. 2008 Jul 16;3(7):e2657. doi: 10.1371/journal.pone.0002657 (PMC2481302; doi:10.1371/journal.pone.0002657)

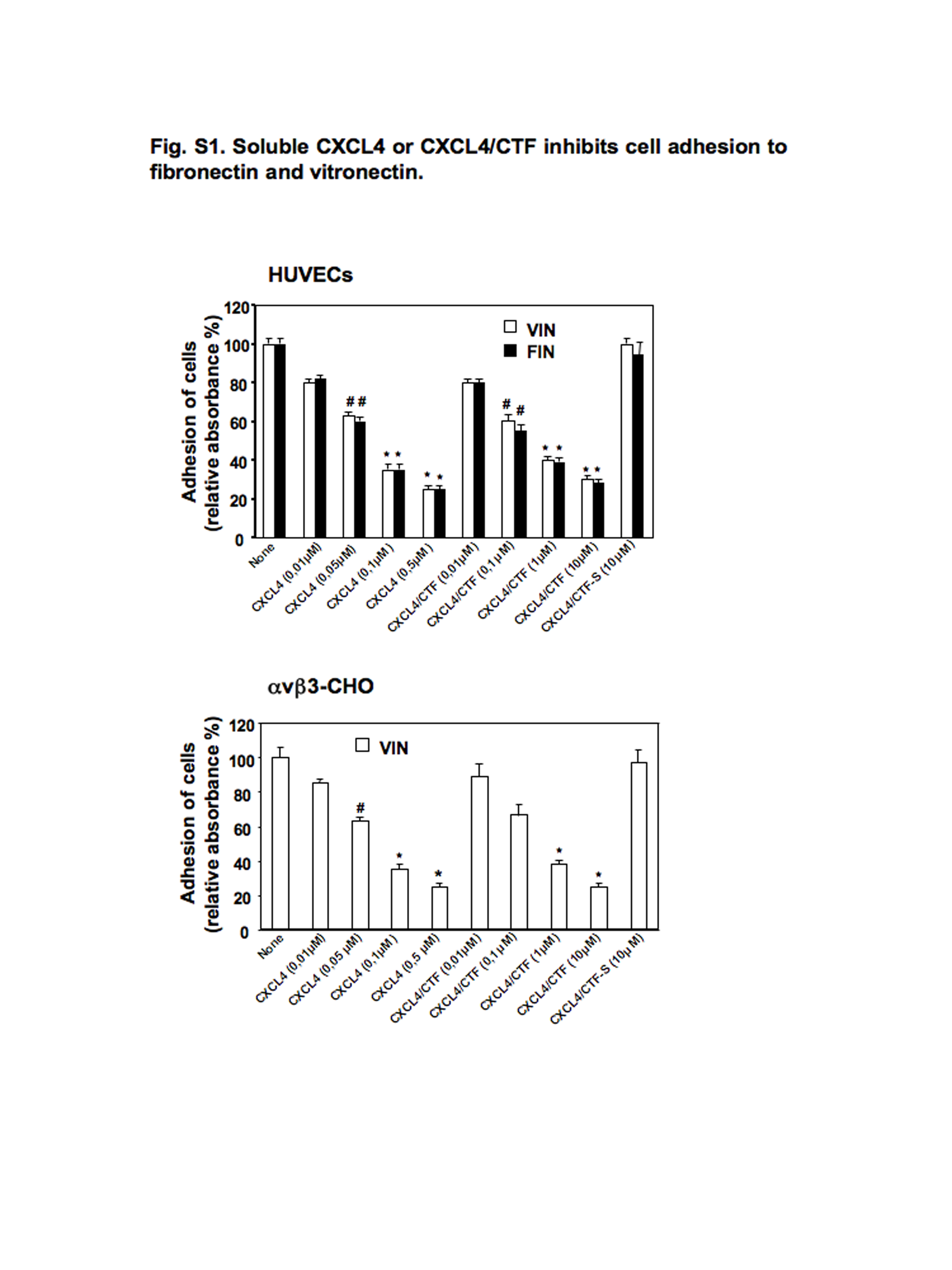

Supplement: Figure S1 — Soluble CXCL4 or CXCL4/CTF inhibits cell adhesion to fibronectin and vitronectin. HUVECs and αvβ3-CHO adhesion on immobilized (10 µg/ml) fibronectin or (10 µg/ml) vitronectin was determined in the presence or absence of the indicated concentrations of CXCL4 or CXCL4/CTF or CXCL4/CTF-S as described in Material and Methods. Error bars represent the mean+SD, #, P<0.005; *, P<0.001 compared to the cell adhesion in the absence of CXCL4 or CXCL4/CTF; n = 2 independent experiments. (39.08 MB DOC) [file pone.0002657.s001.tif]
